# Weibull Parameter Estimation Using Empirical and AI Methods: A Wind Energy Assessment in İzmir

**DOI:** 10.3390/biomimetics10100709

**Published:** 2025-10-20

**Authors:** Bayram Köse

**Affiliations:** Faculty of Engineering and Architecture, Department of Electrical and Electronics Engineering, Izmir Bakırçay University, 35665 Menemen, Izmir, Turkey; bayram.kose@bakircay.edu.tr

**Keywords:** Weibull distribution parameters, Genetic Algorithm (GA), Gravity Search Algorithm (GSA), Sine Cosine Algorithm (SCA), Teaching Learning Algorithm (TLBA), Gray Wolf Algorithm (GWA), Red Fox Algorithm (RFA), red panda optimization algorithm (RPA), wind energy potential

## Abstract

This study evaluates the estimation of Weibull distribution parameters (shape, *k*; scale, *c*) for wind speed modeling in wind energy potential assessments. Traditional empirical methods—Justus Moment Method (JEM), Power Density Method (PDM), Energy Pattern Factor Method (EPFM), Lysen Moment Method (LAM), and Standard Deviation Empirical Method (SEM)—are compared with advanced artificial intelligence optimization algorithms (AIOAs), including Genetic Algorithm (GA), Gravitational Search Algorithm (GSA), Sine Cosine Algorithm (SCA), Teaching-Learning-Based Optimization (TLBA), Grey Wolf Optimizer (GWA), Red Fox Algorithm (RFA), and Red Panda Optimization Algorithm (RPA). Using hourly wind speed data from Foça, Urla, Karaburun, and Çeşme in Turkey, the analysis demonstrates that AIOAs, particularly GA, GSA, SCA, TLBA, and GWA, outperform empirical methods, achieving low RMSE (0.0071) and high R^2^ (0.9755). SEM and LAM perform competitively among empirical methods, while PDM and EPFM show higher errors, highlighting their limitations in complex wind speed distributions. The study also conducts a techno-economic analysis, assessing capacity factors, unit energy costs, and payback periods. Foça and Urla are identified as optimal investment sites due to high energy yields and economic efficiency, whereas Çeşme is unviable due to low production and long payback periods. This research provides a robust framework for Weibull parameter estimation, demonstrating AIOAs’ superior accuracy and offering a decision-support tool for sustainable wind energy investments.

## 1. Introduction

As the global economy and urbanization continue to expand, energy demand is simultaneously rising. The depletion of primary energy resources, recurrent energy crises, environmental challenges, and the increasing severity of global climate change threaten the sustainability of human living standards. In response, governments worldwide are accelerating the integration of renewable and sustainable energy resources into power systems, recognizing the urgent need for global energy conservation and emission reduction [1].

The growing demand for sustainable energy has intensified the adoption of renewable sources such as solar and wind power. Among these, wind energy stands out as a particularly attractive alternative due to its environmentally friendly nature and independence from fuel costs, providing an effective solution against pollution and climate-related challenges [2]. However, the intermittent and stochastic behavior of wind introduces significant variability in power generation, which may adversely affect system reliability, stability, design, and overall cost [2,3]. Thus, accurately characterizing wind regimes is critical for maximizing the potential of wind energy, which is increasingly perceived as a clean and economically viable option in both developed and developing nations [4].

Prior to large-scale wind energy investments, it is essential to assess the wind characteristics and potential of the target site. To this end, several probability density functions (PDFs) are employed to model wind speed distributions, including Weibull, Rayleigh, Gamma, Lognormal, Logistic, Inverse Gaussian, Maximum Entropy Principle (MEP), Pearson type V, Burr, and hybrid functions such as the Gamma–Weibull distribution [5,6]. In addition, a wide range of numerical estimation techniques—such as the Graphical Method (GM), Method of Moments (MM), Least Squares Method (LSM), Maximum Likelihood Method (MLM), Generalized Maximum Likelihood Method (GMLM), and the Energy Pattern Factor Method (EPFM) are used to estimate distribution parameters [1,7].

Recent advances in artificial intelligence (AI) have introduced metaheuristic optimization algorithms as powerful tools for parameter estimation in wind speed modeling. Many of these algorithms are inspired by natural phenomena and swarm intelligence behaviors. For example, Particle Swarm Optimization (PSO) mimics the foraging dynamics of birds and fish, Ant Colony Optimization (ACO) is derived from food-searching behaviors of ant colonies, and Cuckoo Search (CS) is inspired by brood parasitism behavior in cuckoos [1]. Such methods have shown promise in optimizing Weibull distribution function (Wpdf) parameters with higher accuracy compared to conventional techniques [8,9].

Kumar et al. (2019) [10] used the Multiverse Optimization (MVO) method in the study for the Tirumala region of India. The studies have demonstrated the superior capability of metaheuristic algorithms in estimating Weibull distribution parameters for wind energy assessment. In [11], four techniques—Migrating Birds Optimization (MBO), Imperialist Competitive Algorithm (ICA), Cuckoo Search (CS), and Harmony Search (HS)—were applied to model wind speed distributions in two Brazilian regions. Similarly, [12] compared Grey Wolf Optimization (GWO), Particle Swarm Optimization (PSO), and Cuckoo Search (CS) with four numerical approaches for large wind farms in China. According to Samal’s work, the convergence times or running times of the algorithms vary depending on the chosen distribution. The PSO and DE algorithms are significantly faster than the TLBO and ABC algorithms [13].

In related literature, numerous studies have explored these approaches. Genç et al. employed the two-parameter Weibull distribution to characterize wind power potential in Turkey’s Akşehir region, using Maximum Likelihood, Least Squares, and Method of Moments for parameter estimation. Their findings highlighted consistency between empirical data and modeled results, enabling robust potential assessments [14]. Similarly, other studies compared six methods—Graphical, Moment, Energy Pattern Factor, Mean Standard Deviation, Power Density, and Genetic Algorithm—using wind data from İstanbul’s Çatalca region, demonstrating the superior performance of GA and GM in terms of MAE, RMSE, and R^2^ metrics [15].

In Egypt, Zafarana and Shark El-Ouinate wind sites were analyzed using four analytical methods (MLM, MM, EM, and EPFM) and two metaheuristics (PSO and Aquila Optimizer, AO). Results indicated that AO outperformed all alternatives, achieving the lowest RMSE, MAE, and standard errors, alongside superior convergence and stability [16]. Furthermore, comprehensive evaluations of 14 methods across sites with varying wind regimes revealed that EPFM is most reliable for Weibull parameter estimation, whereas WVM and MoroM were found unsuitable. Importantly, it was shown that Weibull parameters vary with altitude: the shape parameter *k* decreases while the scale parameter *c* increases with hub height [17].

Other works have emphasized the suitability of classical methods such as Maximum Likelihood for parameter estimation in operational wind farms [18], while comparative studies combining five classical methods with five AI-based algorithms (PSO, GA, Differential Evolution, Cuckoo Search, and Social Spider Optimization) found that AI approaches consistently yielded superior parameter estimation performance and more accurate wind energy potential predictions [19]. Such findings underscore the increasing relevance of hybrid and AI-driven methodologies in wind energy assessment.

### Motivation and Contributions

Wind energy assessments in Turkey and worldwide predominantly rely on the estimation of Weibull distribution parameters, which are subsequently used to conduct techno-economic analyses of wind energy production based on classical parameter estimation methods. However, the development of artificial intelligence (AI) optimization algorithms has significantly enhanced the ability to optimize Weibull parameters and represent wind speed data with greater accuracy. This advancement has markedly improved the precision of wind energy assessments.

In this study, a comprehensive technical and economic feasibility analysis is carried out for four different wind sites in İzmir. For the first time in this field, beyond conventional empirical approaches, the performance of two newly developed nature-inspired AI optimization algorithms, Red Panda and Red Fox, is evaluated. The study compared the effectiveness of five empirical methods and seven AI-based optimization algorithms (AIOAs) in estimating Weibull parameters, while also examining six wind turbine technologies with varying rated powers and characteristics.

It has been shown that while empirical methods provide baseline estimates, AI-based approaches consistently yield more reliable outputs with respect to the actual wind speed frequency distributions. This is particularly evident in the accurate determination of Weibull parameters, which in turn facilitated robust energy output predictions and optimal turbine selection recommendations for each site.

The research framework integrated both the statistical and optimization perspectives, combining wind resource assessment with techno-economic evaluation of turbines. Specifically, the process included:

1. Identifying candidate sites and collecting wind data,
2. Preprocessing data to address gaps and anomalies,
3. Performing descriptive statistical analyses,
4. Estimating Weibull parameters through empirical and AI-based methods,
5. Validating results using probability density functions and annual energy output calculations,
6. Selecting optimal turbines and computing capacity factors, and
7. Calculating the cost of energy and payback periods.

**Figure 1:**
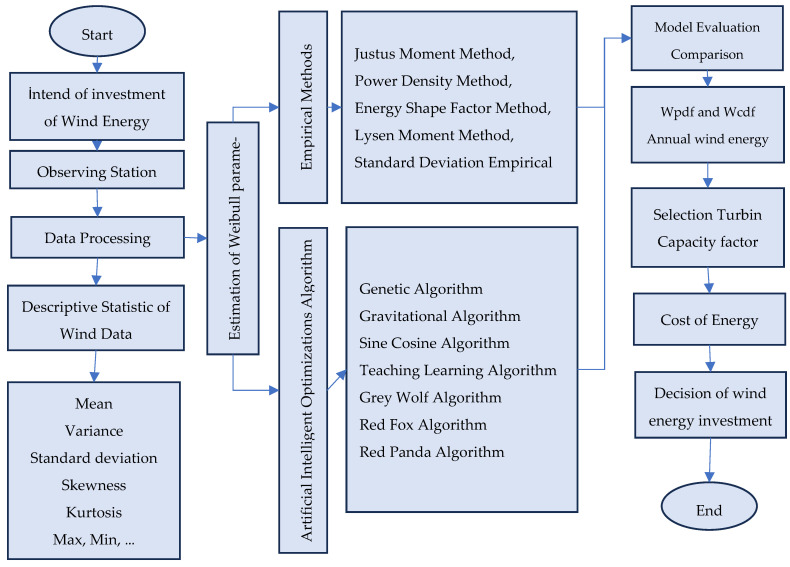
The study framework.

Overall, the proposed framework (Figure 1) not only compares parameter estimation approaches but also offers a decision-support mechanism for wind energy investments in the İzmir region. By combining classical empirical techniques with modern AI-based optimization, the study provides new insights into the accuracy, reliability, and economic implications of wind energy potential assessments.

To accurately determine the parameters of the probability distribution function, a total of twelve estimation methods were employed, comprising five empirical methods (EMs) and seven metaheuristic optimization algorithms. The Weibull distribution model is subsequently constructed, and its performance is rigorously evaluated using statistical goodness-of-fit indicators, including the Root Mean Squared Error (RMSE) and the Coefficient of Determination (R^2^). These criteria enabled a robust comparison of the accuracy and reliability of different parameter estimation approaches. Furthermore, comparative techno-economic feasibility analyses are conducted for the selected sites, integrating technical indicators such as capacity factor and annual energy production with economic measures such as cost of energy (COE) and payback period. This comprehensive framework not only validates the accuracy of the models but also provides actionable insights for wind energy resource assessment and investment decision-making.

## 2. Methodology

### 2.1. Data

In this study, wind speed data were obtained from long-term measurements conducted at four meteorological stations—Foça (Azaplar), Urla, Karaburun, and Çeşme—operated by the Turkish State Meteorological Service in the İzmir region. The dataset comprises hourly average wind speeds derived from 10 min interval recordings over extended observation periods. Prior to analysis, a comprehensive data pre-processing procedure was applied to ensure reliability and consistency. This included the removal of erroneous measurements caused by sensor malfunctions, elimination of duplicate records, interpolation of short-term missing data (e.g., several-hour gaps), and exclusion of periods with significant data loss. After pre-processing, the final dataset consisted of 8594 h of observations for Foça, 8710 h for Urla, 8107 h for Karaburun, and 8718 h for Çeşme. The curated dataset ensures high data integrity and enhances the reproducibility of subsequent analyses. Fundamental statistical descriptors and wind occurrence frequencies for each site are summarized in Table 1 and Table 2, providing essential insights into the wind characteristics and distribution patterns of the study regions.

Table 1 provides a summary of the principal statistical measures of the wind speed series for all sites, including mean, median, standard deviation, skewness, kurtosis, range, maximum, minimum, and the number of observations. These descriptors offer critical insights into the underlying wind regime of each location. The mean and median values reflect the central tendency of wind speeds, while the standard deviation highlights the variability and dispersion of the data. Skewness is particularly important, as it quantifies the degree of asymmetry in the wind speed distribution, thereby indicating whether the distribution is biased towards higher or lower wind speeds. Similarly, kurtosis provides information about the “peachiness” and tail behavior of the distribution, which is crucial for assessing the likelihood of extreme wind events.

By incorporating these measures, the analysis captures not only the average characteristics of wind regimes but also the variability, asymmetry, and occurrence of extremes, which directly influence energy yield estimates and the reliability of wind power projects. These statistical evaluations thus form the foundation for accurate parameter estimation of probability density functions, such as the Weibull distribution, and provide the basis for subsequent techno-economic assessments of wind energy potential.

The average wind speed and standard deviation were calculated from the measured data using Equations (1) and (2).

(1) $$\bar{v}=\frac{1}{n}\sum_{i=1}^{n}v_{i}$$

In Equation (1), *n* represents the number of data and *v_i_* is the *i*-th measured wind speed data. *σ* is the value of the standard deviation of the measured wind speeds and is computed by Equation (2)

(2) $$\sigma ={\left[\frac{1}{n-1}\sum_{i=1}^{n}{\left(v_{i}-\bar{v}\right)}^{2}\right]}^{0.5}$$

Skewness is a statistical measure that describes the degree of asymmetry of a distribution around its mean. A positive skewness value indicates a distribution with a longer tail extending toward higher positive values, whereas a negative skewness value reflects a distribution with a longer tail extending toward lower negative values. When skewness approaches zero, the distribution tends to become more symmetric. The skewness coefficient (SK) is defined by Equation (3) [20].

(3) $$SK=\frac{1}{\left(n-1\right)}\sum {\left(\frac{v_{i}-\bar{v}}{\sigma }\right)}^{3}$$

Here, n is the number of observations, $\bar{v}$ is the mean wind speed, and σ denotes the standard deviation. As shown in Table 1, the highest skewness is observed at the Çeşme station, while all sites exhibited positive skewness values, indicating a general tendency of wind speed distributions to extend toward higher values.

Kurtosis, on the other hand, characterizes the relative peakedness or flatness of a distribution compared to the normal distribution. A positive kurtosis value indicates a more peaked distribution, while a negative kurtosis value suggests a flatter shape [20]. The kurtosis coefficient (KR) is given in Equation (4):

(4) $$KR=\frac{1}{n-1}\sum {\left(\frac{v_{i}-\bar{v}}{\sigma }\right)}^{4}-3$$

According to Table 1, all stations except Urla demonstrated positive kurtosis values, implying a more peaked distribution, whereas Urla exhibited negative kurtosis, indicating a relatively flatter distribution.

Table 2 presents the wind speed frequency distributions for each location, covering a range from 0 m/s to 23 m/s. These frequencies represent the relative occurrence of wind speeds within specific intervals and provide insights into the probability of different wind regimes at each site. Such information is essential for accurately characterizing wind resources and assessing their suitability for energy production.

**Figure 2:**
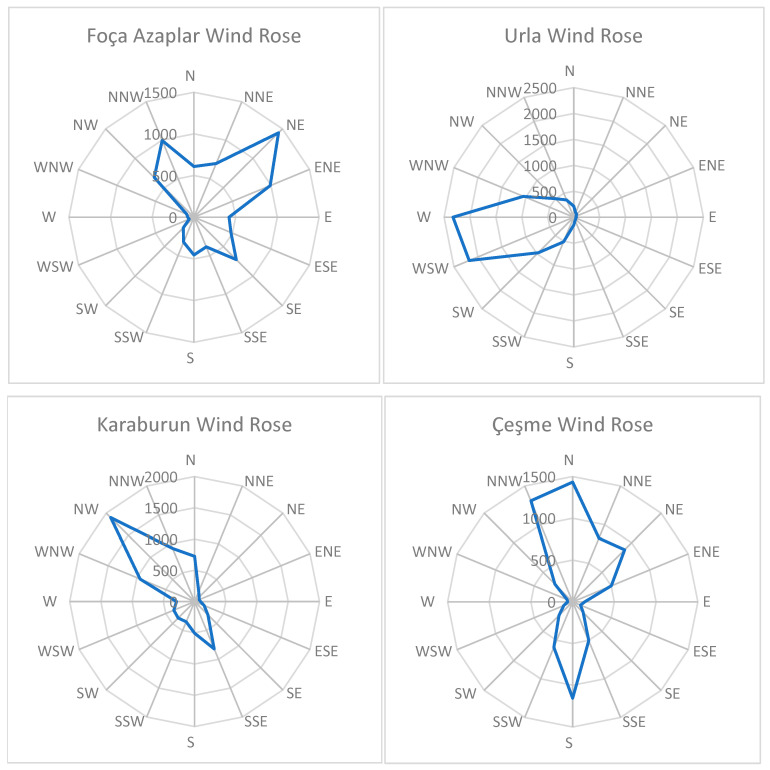
Wind rose charts for observation stations.

The wind rose diagram, divided into sixteen directional sectors, is presented in Figure 2 to illustrate the prevailing wind directions. These diagrams provide a visual representation of the frequency and intensity of winds originating from different directions. For each location, wind rose plots were employed to characterize wind regimes and to identify the prevailing wind directions. The dominant wind directions for each district are summarized in Figure 2.

The graphical analysis highlights how wind data are visually represented and emphasizes the wind characteristics specific to each site. According to the prevailing wind rose patterns shown in Figure 2, the dominant wind direction in Foça (Azaplar) is observed to be from the northeast and north-northwest sectors. In Urla, the prevailing winds predominantly originate from the west. For Karaburun, the dominant flow is from the northwest and south-southeast directions. Finally, in Çeşme, the winds primarily blow along the north–south axis.

### 2.2. Weibull Probability Distribution Function

The Weibull distribution function (Wpdf) is one of the most widely employed statistical models for characterizing wind speed frequency distributions. Its parameters can be reliably estimated from measured wind speed data using various statistical methods. The probability density function (PDF) of the Weibull distribution is expressed in Equation (5), while the cumulative distribution function (CDF) is given in Equation (6) [21].

(5) $$f\left(v\right)=\frac{k}{c}{\left(\frac{v}{c}\right)}^{k-1}exp\left(-{\left(\frac{v}{c}\right)}^{k}\right)$$

(6) $$F\left(v\right)=1-exp\left(-{\left(\frac{v}{c}\right)}^{k}\right)$$

where *k* is the dimensionless shape parameter, and *c* is the scale parameter in (m/s). These parameters jointly determine the distribution profile and are critical for assessing wind resource potential.

### 2.3. Parameter Estimation Methods

Accurate estimation of Weibull parameters is essential for wind speed characterization and subsequent wind energy assessments. In this study, five empirical methods are considered for parameter estimation and performance comparison. Commonly applied techniques include the Maximum Likelihood Method (MLM), the Method of Moments (MM), and the Least Squares Method (LSM), among others [9]. These approaches have been extensively used in the literature to evaluate the suitability of Weibull models under diverse wind regimes, thereby providing a reliable framework for assessing wind energy potential and turbine performance [9,22].

In addition to classical statistical approaches, the estimation of probability distribution function parameters has increasingly relied on artificial intelligence (AI)–based optimization algorithms. Among the most widely applied are Particle Swarm Optimization (PSO), Genetic Algorithms (GA), and Cuckoo Search Algorithm (CSA) [19]. These algorithms are capable of providing parameter estimates that are remarkably close to actual observed values, thereby improving the accuracy of wind speed distribution modeling [9]. In this study, seven distinct AI optimization algorithms are employed, two of which, according to the literature survey, are applied for the first time in this context [15,23]

### 2.4. Empirical Methods for Wpdf Parameter Estimation

#### 2.4.1. Moment Method (MM)

In this study, the Weibull parameters *k* (shape factor) and *c* (scale factor) were also estimated using the Moment Method (MM), often referred to as the Justus Empirical Method (JEM). This approach utilizes the statistical mean and standard deviation of the wind speed data. The mathematical formulations are given in Equations (7) and (8), where the method directly relates the first and second central moments of the observed data to the Weibull distribution parameters [24].

(7) $$k={\left(\frac{\sigma }{\bar{v}}\right)}^{-1.086}$$

(8) $$c=\frac{\bar{v}}{\Gamma \left(1+\frac{1}{k}\right)}$$

#### 2.4.2. Energy Pattern Factor Method

Akdağ and colleagues employ the energy pattern factor (EPF, $E_{pf}$), defined as the ratio of the mean cube of wind speeds to the cube of the mean wind speed. This parameter is then used to estimate the Weibull shape and scale parameters through empirical relationships [25]. The energy pattern factor is calculated as:

(9) $$E_{pf}=\frac{\bar{v^{3}}}{{\bar{v}}^{3}}=\frac{\sum_{i=0}^{n}f_{i}v_{i}^{3}}{{\left(\sum_{i=0}^{n}f_{i}v_{i}\right)}^{3}}=\frac{\Gamma \left(1+\frac{3}{k}\right)}{\Gamma^{3}\left(1+\frac{1}{k}\right)}$$

Subsequently, the Weibull parameters are obtained using:

(10) $$k=3.957\cdot {\left(E_{pf}\right)}^{-0.898}$$

(11) $$c=\frac{\bar{v}}{\Gamma \left(1+\frac{1}{k}\right)}\;$$

#### 2.4.3. Power Density Method

In the power density method (PDM), the $E_{pf}$ given in Equation (9) is first calculated, and this formula is substituted into Equation (12) to calculate k  [24]. This resulting shape parameter c is calculated using the scale parameter formula given in Equation (11).

(12) $$k=1+\frac{3.69}{{\left(E_{pf}\right)}^{2}}$$

#### 2.4.4. Lysen Empirical Method (LEM)

The Lysen Empirical Method relies on the mean wind speed and its standard deviation. Here, the shape parameter k is calculated using Equation (9), whereas the scale parameter *c* is determined through the following relationship [25].

(13) $$c=\bar{v}{\left(0.568+\frac{0.433}{k}\right)}^{-\frac{1}{k}}$$

#### 2.4.5. Standard Deviation Empirical Method (SEM)

The Standard Deviation Empirical Method estimates the Weibull parameters as a function of the arithmetic mean and standard deviation of the wind speed. The corresponding empirical equations are expressed as [25,26]

(14) $$k={\left(\frac{\sigma }{\bar{v}}\right)}^{-1.086}$$

(15) $$c=\frac{\bar{v}\cdot k^{2.6674}}{0.184+0.816\cdot k^{2.73855}}$$

### 2.5. Optimization Algorithms for Wpdf Parameter Estimation

Metaheuristic optimization algorithms are nature-inspired techniques designed to efficiently explore complex search spaces. In this study, the selected algorithms were employed to minimize the discrepancy between the observed wind speed frequency distribution and the theoretical values obtained from probability distribution functions (PDFs) [9,27]. The objective function used for parameter estimation is defined in Equation (16):

(16) $$Obj.\;func.\left(v_{i}\right)=\sum_{v=0}^{v_{max}}{\left(f_{a}\left(v\right)-f_{pdf}(v)\right)}^{2}$$

where $f_{a}\left(v\right)$, represents the observed frequency distribution for a given wind speed class, $f_{pdf}(v)$ denotes the theoretical values derived from the PDFs, and *n* indicates the total number of wind speed class midpoints.

In order to ensure consistency across methods, the population size was fixed at 50 for all optimization algorithms, and the maximum number of iterations was set to 1000. The search space boundaries for Weibull parameter estimation were defined within the range [0, 23]. Detailed descriptions of the applied artificial intelligence optimization algorithms are presented in this section. To evaluate the accuracy of Weibull parameter estimation, seven metaheuristic optimization algorithms were implemented in this study. These algorithms were selected because of their proven efficiency in solving nonlinear, multimodal optimization problems, and their adaptability to stochastic wind speed distributions.

Inspired by the principles of natural selection and genetics, GA employs operations such as crossover, mutation, and selection to explore the search space efficiently. Its robustness in handling nonlinear and complex objective functions makes it a widely used benchmark algorithm. Modeled after the social behavior of birds and fish, PSO adjusts candidate solutions (particles) by updating their velocities and positions according to both individual and collective best experiences. Its strength lies in fast convergence toward global optima. Based on trigonometric functions, SCA updates candidate solutions along sine and cosine trajectories, providing an effective balance between exploration and exploitation.

This algorithm mimics the interaction between a teacher and learners in a classroom environment. Candidate solutions are iteratively updated according to the knowledge transfer from the best-performing solution (teacher) and the mutual learning among peers. Inspired by the leadership hierarchy and cooperative hunting strategies of grey wolves, GWA divides the population into alpha, beta, delta, and omega wolves, which collaboratively guide the search process. Recently developed and nature-inspired, this algorithm simulates the predatory strategies and adaptive behaviors of red foxes in their natural habitat. To the best of our knowledge, this is among the first applications of RFA for Weibull parameter estimation. Another newly introduced nature-inspired method, RPA imitates the exploration and exploitation behaviors of red pandas, particularly their foraging and climbing skills. Similar to RFA, this study represents one of the earliest attempts to apply RPA in wind energy modeling.

#### 2.5.1. Genetic Algorithm (GA)

**Figure A1:**
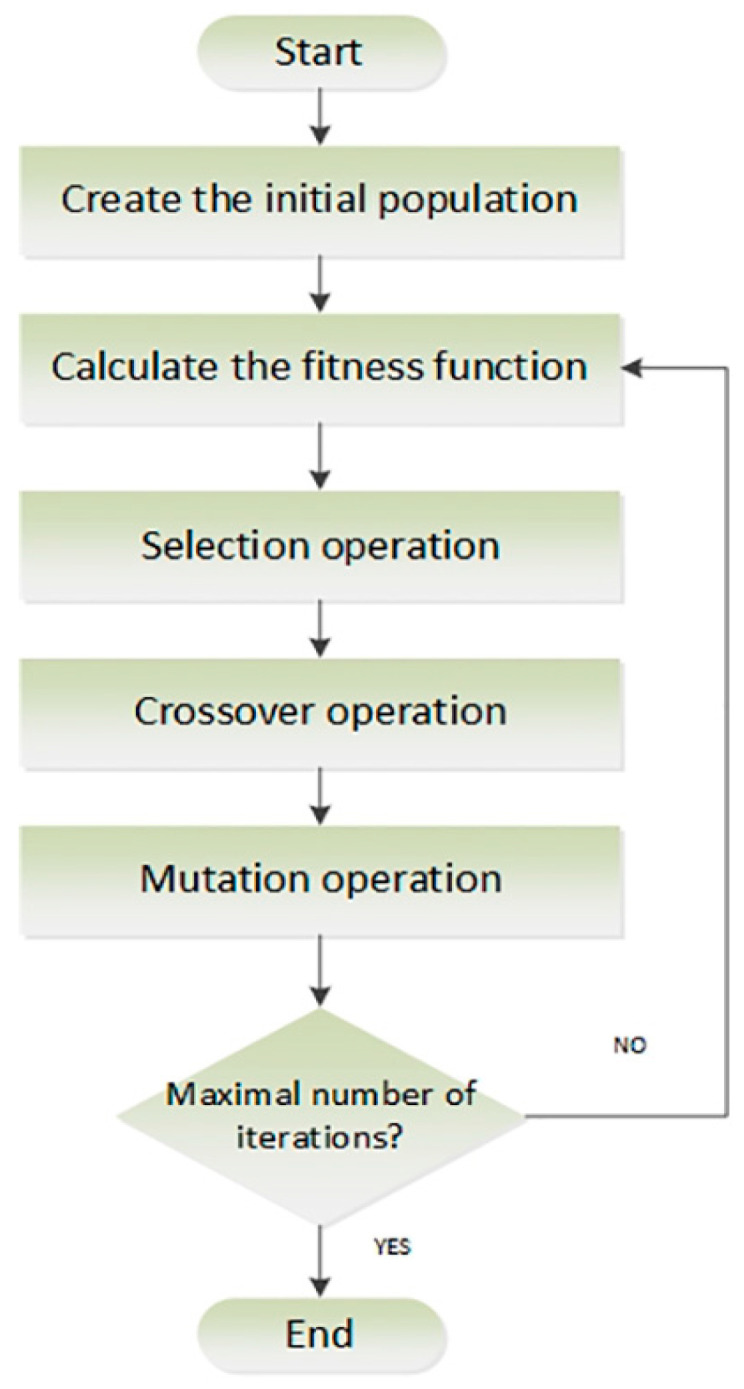
GA flowchart [46].

GA is derived from the actions taken by living organisms in their natural lives to reproduce and survive on a cellular and individual level. GA is a natural selection-based algorithm that uses a population-based approach. The flow chart showing the operational framework of the GA’s optimal solution is given in Figure A1. Selection, crossover, and mutation are the three basic operations of GA. With the selection operator, the best individuals from the old population are moved straight to the new population to be formed at a set pace. Individuals containing the genes (chromosomes) are chosen from random places and brought together in the crossover operator, and an even number of individuals in the crossover pool are randomly matched. New individuals with significant hereditary features are created in this way. Finally, the mutation operator performs a controlled search without becoming stuck at local points in the solution space with random changes in the genes that comprise the individuals [28,29].

#### 2.5.2. Gravitational Search Optimization Algorithm (GSO)

**Figure 3:**
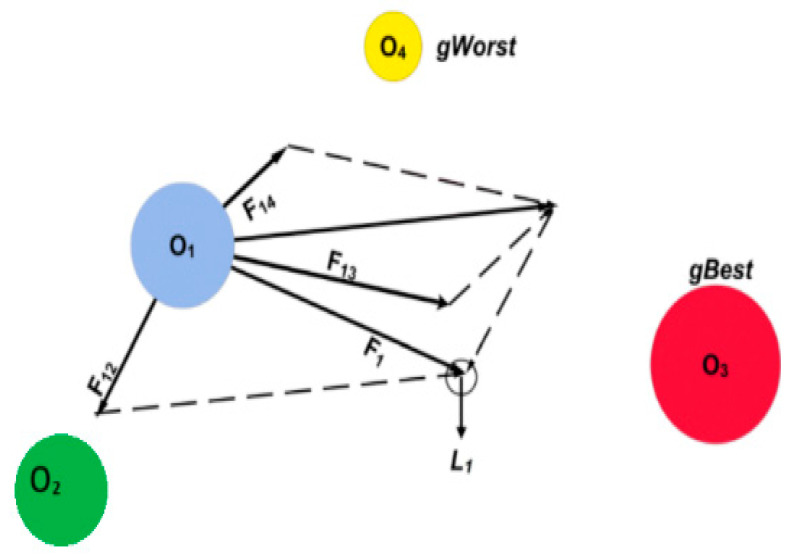
Interaction of masses with gravity and the law of motion.

The Gravitational Search Optimization (GSO) algorithm, proposed by Rashedi et al. in 2009 [30], is a physics-inspired metaheuristic that draws upon Newton’s laws of motion and universal gravitation. In this approach, agents are modeled as objects with masses, where the mass of each object reflects its performance in solving the optimization problem. Objects attract each other with gravitational forces, and the heavier masses exert stronger pulls on lighter ones, guiding the search process toward promising regions of the solution space [30]. A schematic diagram showing the interaction and motion of masses with gravity is provided in Figure 3. Each object’s position in a d-dimensional search space is defined as

(17) $$x_{i}=(x_{i}^{1}\ldots \ldots x_{i}^{d})\;için\;i\;=\;1,\;2,\ldots \ldots \;N$$

where N is the total number of agents, and $x_{i}^{d}$ denotes the position of the *i*-th agent in dimension d. The gravitational constant G decreases over time to balance exploration and exploitation:

(18) $$G(t)=G_{o}.e^{-\propto \frac{t}{T}}$$

where $G_{0}$ is the initial gravitational constant, *α* is a decay parameter, *t* is the iteration number, and *T* is the maximum number of iterations [30].

At each iteration, the best and worst fitness values are defined as:

(19) fbest(t)=maxfiti(t)i=1nmass

(20) fworst (t)=minfiti(t)i=1nmass

Here, $fit_{i}(t)$ denotes the fitness of the i-th agent at iteration t. The active, passive, and inertial masses of agents are computed as:

(21) $$M_{ai}=M_{pi}=M_{ii}=M_{i}$$

(22) $$m_{i}=\frac{{fit}_{i}(t)-f_{worst}(t)}{f_{best}(t)-f_{worst}(t)}$$

(23) $$M_{i(t)}=\frac{m_{i}(t)}{\sum_{j=1}^{N}m_{j}(t)}$$

The Euclidean distance $R_{ij}(t)$ between two agents is expressed as:

(24) $$R_{ij}(t)={\left\|x_{i}(t),x_{j}(t)\right\|}_{2}$$

and the gravitational force exerted on agent  i in dimension d by agent j is given by:

(25) $$F_{ij}^{d}(t)=G(t)\frac{M_{pi}\left(t\right)\times M_{ai}\left(t\right)}{R_{ij}\left(t\right)+\varepsilon }{(x}_{j}^{d}(t)-x_{i}^{d}(t))$$

where *ε* is a small constant to prevent division by zero. The acceleration of each agent is then determined by:

(26) $$a_{i}^{d}(t)=\frac{F_{i}^{d}(t)}{M_{ii}(t)}$$

(27) $$a_{i}\left(t\right)=G\left(t\right)\sum_{j=1}^{n_{mass}}r_{j}\frac{M_{j}\left(t\right)}{R_{ij}\left(t\right)+\varepsilon }\left(x_{j}-x_{i}\right)$$

and its velocity and position are updated as:

(28) $$V_{i}^{d}(t+1)=r_{i}\times V_{i}^{d}(t)+a_{i}^{d}(t)$$

(29) $$x_{i}\left(t+1\right)=x_{i}(t)+v_{i}(t+1)$$

**Figure A2:**
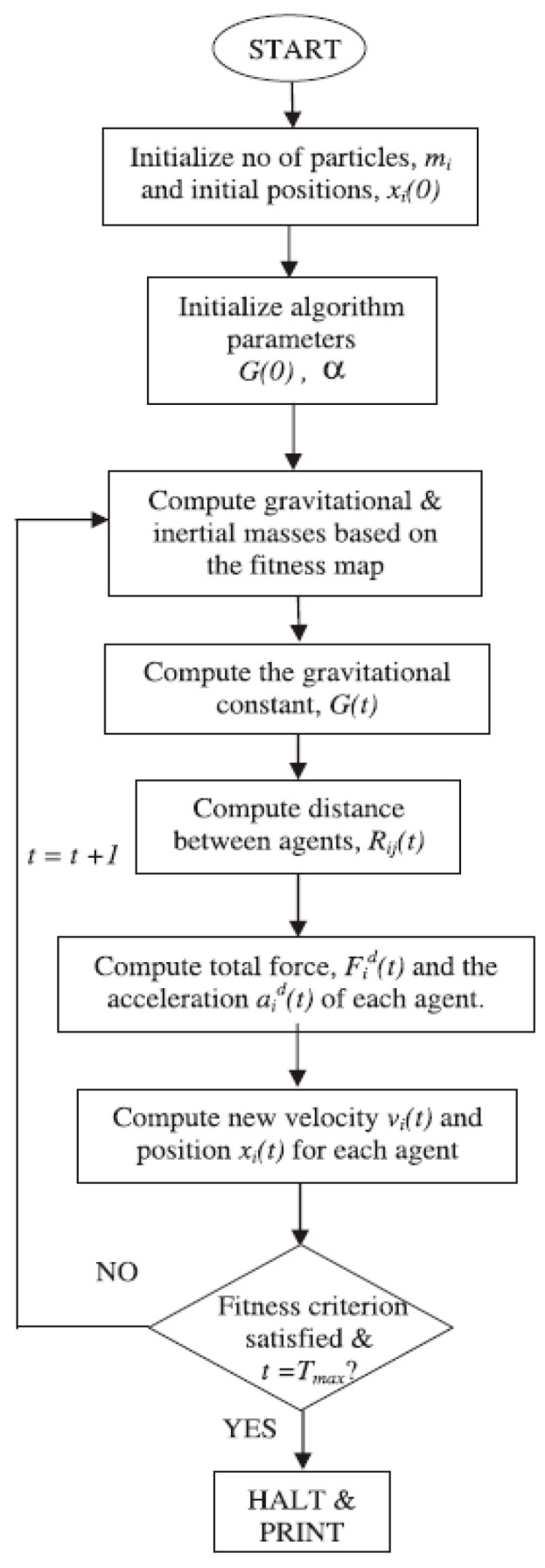
GSA flowchart [31].

Here, $r_{i}$ ∈ [0, 1] is a uniformly distributed random number. The algorithm terminates when the maximum number of iterations is reached, or a predefined stopping criterion is satisfied. The GSA workflow diagram is shown in Figure A2. By simulating gravitational interactions among agents, GSO provides a powerful mechanism for solving complex, high-dimensional optimization problems [30,31,32].

#### 2.5.3. Sine Cosine Optimization Algorithm (SCA)

The Sine Cosine Algorithm (SCA) is a population-based metaheuristic optimization technique. At its core, the algorithm initializes multiple random candidate solutions and iteratively updates them either toward or away from the current best solution. To balance exploration (global search) and exploitation (local refinement), several random and adaptive control parameters are incorporated. This balance is crucial, as exploration identifies promising regions in the search space, while exploitation ensures convergence toward the global optimum [33].

During the exploitation phase, stochastic variations applied to candidate solutions are relatively small compared to the exploration phase, enabling fine-tuning around the best solutions. The position updating mechanism of SCA is governed by sine and cosine mathematical functions, as shown in Equations (30) and (31):

(30) $$X_{i}^{t+1}=x_{i}^{t}+r_{1}\times sin\left(r_{2}\right)\times \left|r_{3}P_{i}^{t}-X_{i}^{t}\right|$$

(31) $$X_{i}^{t+1}=x_{i}^{t}+r_{1}\times cos\left(r_{2}\right)\times \left|r_{3}P_{i}^{t}-X_{i}^{t}\right|$$

where $x_{i}^{t}$ is the current solution of the i.-th candidate at iteration t; $P_{i\;}$ is the target position; $r_{1},\;r_{2},\;r_{3}$ are random numbers; and $r_{4\;}$ ∈ [0, 1] determines whether sine or cosine will be applied in each update. These equations define the movement of solutions across the search space, with sine and cosine functions introducing oscillatory dynamics that allow both inward and outward movements relative to the target [33].

To unify the sine and cosine updates, Equation (32) is used:

(32) $$X_{i}^{t+1}=\left\{\begin{array}{c} \;x_{i}^{t}+r_{1}\times \sin\left(r_{2}\right)\times \left|r_{3}P_{i}^{t}-X_{i}^{t}\right|,\;{\;r}_{4\;}<0.5\;\\ x_{i}^{t}+r_{1}\times \cos\left(r_{2}\right)\times \left|r_{3}P_{i}^{t}-X_{i}^{t}\right|,\;{\;r}_{4\;}\geq 0.5 \end{array}\right.$$

The four main parameters of SCA play distinct roles:

$r_{1}:$ Determines the region of movement for the next position.

$r_{2}$: Controls the direction and step size toward or away from the target.

$r_{3}$: Introduces stochastic weighting; values greater than 1 emphasize exploration, while values less than 1 promote exploitation.

$r_{4}$: Controls the switch between sine and cosine operators.

To ensure dynamic exploration and exploitation, the range of sine and cosine functions is adaptively decreased over iterations, as given in Equation (33):

(33) $$r_{1}=a-t\frac{a}{T}$$

where t is the current iteration, *T* is the maximum number of iterations, and *a* is a constant. This mechanism allows broad exploration during early iterations and gradually shifts toward exploitation in later stages.

**Figure 4:**
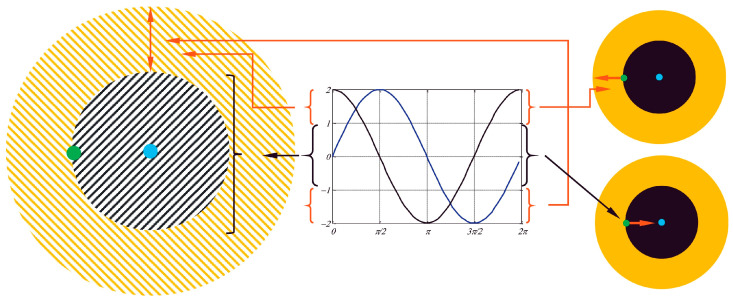
Navigating Around or Beyond the Solution of the SCA within a Certain Range.

**Figure A3:**
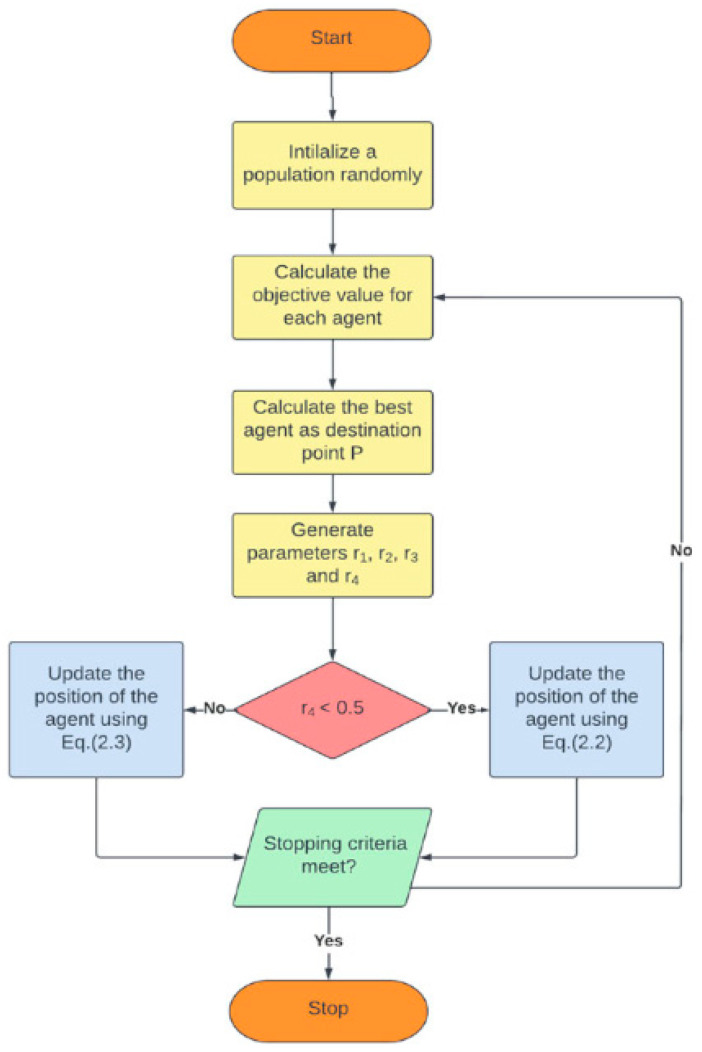
The flowchart of SCA [35].

The overall flow of SCA begins with the random initialization of solutions (Figure A3), followed by iterative updates based on the sine–cosine operators. The best solution found so far is preserved and designated as the guiding target for the population. The algorithm terminates when the maximum number of iterations is reached, or a predefined stopping criterion is satisfied. Figure 4 shows the sinus and cosine algorithms adaptive navigation around or beyond the solution within a certain range. By combining oscillatory mathematical dynamics with adaptive parameter control, SCA achieves an effective trade-off between global exploration and local exploitation, making it a competitive optimizer for complex real-world problems [33,34,35].

#### 2.5.4. Teaching Learning Based Optimization Algorithm (TLBA)

The Teaching-Learning-Based Optimization (TLBA) algorithm is a population-based metaheuristic inspired by the interaction between a teacher and students in a classroom environment. In this framework, both the teacher and the students represent potential solutions to the optimization problem, with the teacher symbolizing the current best solution [33]. Each candidate solution is modeled as a series of variables that correspond to a student’s performance across different subjects. Students enhance their performance either through knowledge gained from the teacher (the teacher phase) or through peer-to-peer interaction (the learner phase) [36].

During the teacher phase, students adjust their performance relative to the best individual (teacher) and the class mean, as formulated in Equation (34):

(34) $${\;x}^{i+1}=x^{i}+r(x_{t}-T_{f}x_{ort})$$

here, *T_F_* is the teaching factor, which can take values of 1 or 2 (in this study, *T_F_* = 1), and by *r* is a uniformly distributed random number in the range [0, 1]. The new student solution is generated by improving upon the old solution, while the class mean vector *x^i^* is calculated as:

(35) $$x_{m}=[m\left(x_{1}\right)\ldots m\left(x_{s-1}\right),m\left(x_{n}\right)]$$

In the learner phase, students interact with one another to further improve their performance. Knowledge is transferred from better-performing students to weaker ones, as represented in Equation (36):

(36) $$f\left(x^{i}\right)\;\left\{\begin{array}{cc} x^{yeni,i}=x^{\prime }+r\left(x^{i}-x^{j}\right), & f(x^{i})<f(x^{j})\\ x^{yeni.j}=x^{\prime }+r\left(x^{j}-x^{i}\right),\; & f(x^{i})>f(x^{j})\; \end{array}\right.$$

If the newly generated solution outperforms the previous one, it replaces the older candidate in the population. This two-stage mechanism ensures continuous improvement through both guidance from the best solution and mutual peer learning.

**Figure A4:**
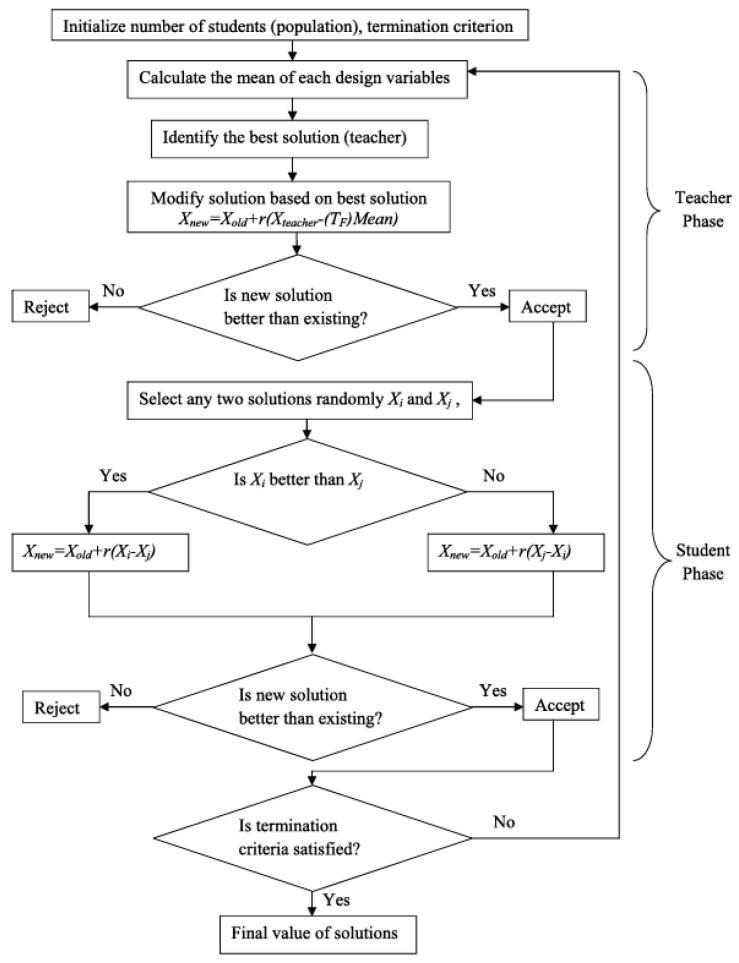
The flowchart for TLBA [37].

Unlike many other metaheuristics, TLBA does not require algorithm-specific control parameters such as crossover or mutation rates. Its only required settings are population size and the maximum number of iterations, which are standard for population-based algorithms. The behavior of the Teaching-Learning algorithm in finding the optimal solution is shown in Figure A4 as a flowchart. This simplicity makes TLBA highly attractive for solving a wide range of optimization problems and has contributed to its increasing popularity in recent years. Comprehensive details regarding the theoretical foundations and applications of TLBA can be found in earlier studies [37,38].

#### 2.5.5. Grey Wolf Optimization Algorithm (GWA)

The Grey Wolf Optimizer (GWA) is a nature-inspired metaheuristic algorithm derived from the mathematical modeling of the social hierarchy and hunting behavior of grey wolves. In this framework, the best solution is denoted as the “Alpha” *α*, the second and third best solutions as “Beta” *β* and “Delta” *δ*, respectively, while the remaining candidate solutions are considered “Omega” *ω*. The optimization process, analogous to the wolves’ hunting strategy, is guided by the three leader wolves (*α*, *β*, *δ*), while the omegas follow their lead [39].

GWA mathematically simulates the encircling mechanism exhibited by wolves during hunting. This behavior is modeled using coefficient vectors *A* and *C*, which dynamically change over iterations. Specifically, vector *A* decreases linearly from 2 to 0, while vector *C* takes random values within the interval [0, 1]. This adaptive mechanism allows wolves to encircle the prey, thereby improving their chances of converging toward promising regions of the search space [40].

During the encircling phase, the position of each wolf is updated relative to the estimated position of the prey, which is determined based on the knowledge of the alpha, beta, and delta wolves. The omega wolves update their positions by following these leaders, thereby enhancing both exploration and exploitation capabilities of the algorithm. This mechanism is formally expressed in Equations (37)–(40):

(37) $$D=\left|C\cdot X_{p}\left(t\right)-X(t)\right|$$

(38) $$X\left(t+1\right)=X_{p}\left(t\right)-A\cdot D$$

(39) $$A=2ar_{1}-a$$

(40) $$C=2r_{2}$$

Here, $X_{p}\left(t\right)$ represents the prey position, $r_{1}$ and $r_{2}$ are random numbers in [0, 1], and a decreases linearly with iterations. The hunting phase incorporates the collective knowledge of the alpha, beta, and delta wolves to approximate the prey’s position more accurately. This is modeled in Equations (41)–(43):

(41) $$D_{1}=\left|{C_{1}X}_{1}-X\right|;D_{2}=\left|{C_{2}X}_{2}-X\right|;D_{3}=\left|{C_{3}X}_{3}-X\right|$$

(42) $$X_{1}=X_{1}-A_{1}D_{1};X_{2}=X_{2}-A_{2}D_{2};X_{3}=X_{3}-A_{3}D_{3}$$

(43) $$X\left(t+1\right)=\frac{X_{1}+X_{2}+X_{3}}{3}$$

**Figure A5:**
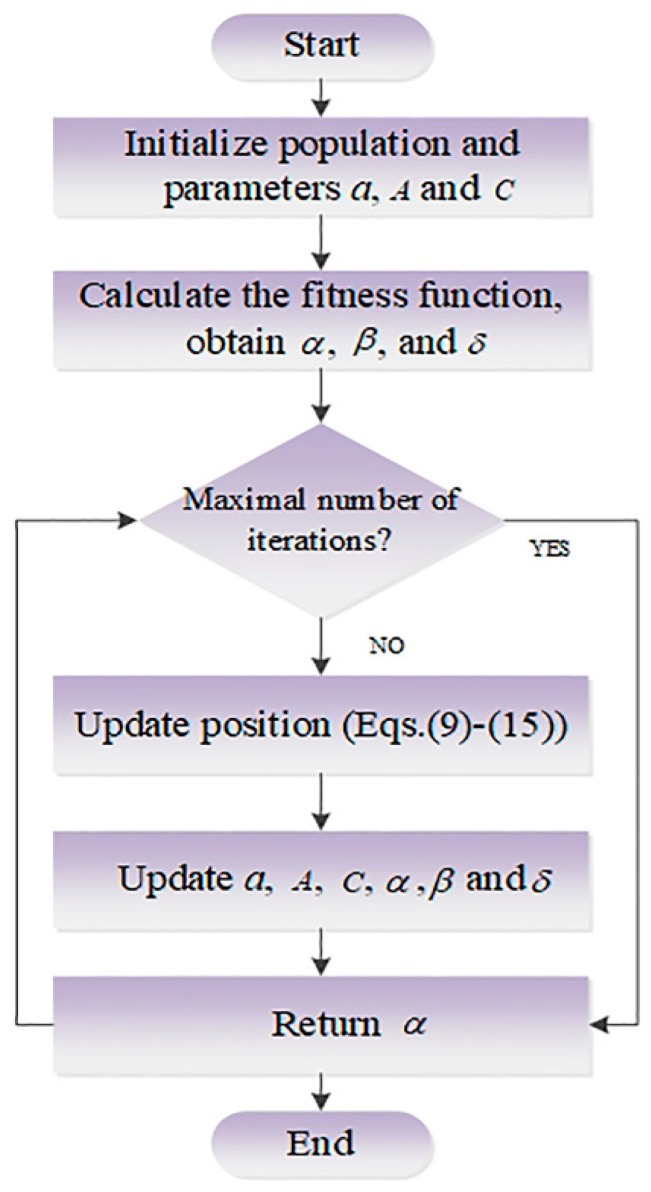
The flowchart for GWA [46].

The simplicity of GWA offers a clear visualization of how wolves encircle and exert pressure on prey, which translates to effective optimization dynamics. By leveraging both exploration (diverse prey search) and exploitation (convergence toward prey), GWA has proven highly effective in solving a wide range of complex optimization problems. The behavior of the gray wolf optimization algorithm in finding the optimal solution is shown in Figure A5 as a flowchart. Its balance between global search and local refinement enhances the likelihood of attaining global optima, making GWA one of the most widely adopted algorithms in recent computational intelligence research.

#### 2.5.6. Red Fox Optimization Algorithm (RFA)

The Red Fox Optimization Algorithm (RFA) is a nature-inspired metaheuristic approach modeled on the behavioral strategies of red foxes. In nature, foxes display versatile survival and hunting techniques, actively engaging in both wild and domestic hunting throughout the year [41]. Alpha pairs typically establish a defined hunting territory, while younger foxes eventually disperse to explore new areas. These juveniles, drawing on the inherited knowledge of their parents, seek and control new hunting grounds. However, some foxes may leave the group entirely, establishing their own independent packs.

When red foxes encounter potential prey during their journeys, they adopt stealthy and concealed approaches, minimizing detection. Each member of the pack plays a vital role in survival; if food resources are scarce in their territory, individuals scatter to search in distant areas. Should these explorations fail, the foxes return to their original habitat. Conversely, if adequate resources are found, the knowledge of these regions is shared with the rest of the group. In RFA, such natural behaviors are simulated to represent exploration, exploitation, and the dynamic balance between them [42]. Within the algorithm, each individual (fox) is represented by a solution vector:

$$x=(x_{1},x_{2},\ldots ,x_{n})$$

During the global search phase, individuals explore distant regions, guided by their Euclidean distance from the current best solution:

(44) $$d\left({\left(x_{i}\right)}^{t},{\left(x_{best}\right)}^{t}\right)=\sqrt{\left\|{\left(x_{i}\right)}^{t}-{\left(x_{best}\right)}^{t}\right\|}$$

New positions are updated toward the best solution using a random scaling factor:

(45) $${\left(x_{i}\right)}^{t}={\left(x_{i}\right)}^{t}+a\cdot sign({\left(x_{best}\right)}^{t}{-\left(x_{t}\right)}^{t})$$

If the new position improves the fitness level, the individual retains it; otherwise, it reverts to its previous location. This mechanism simulates the scenario in which foxes return home when distant searches yield no food. Additionally, the worst-performing individuals in the population are removed, reflecting natural predation or migration pressures.

In the local search phase, foxes mimic stealth hunting behavior. This is controlled by a random parameter μ:

(46) $$\left\{\begin{array}{c} \mu \leq 0.75\;=>\;Waiting\;and\;hiding\\ \mu >0.75\;=>Approaching\;the\;prey \end{array}\right.$$

The approaching behavior is modeled using a cochleoid curve, incorporating parameters such as observation radius, viewing angle, and stochastic environmental conditions (e.g., fog, rain). The observation radius is defined by parameters a∈(0,0.2) and $\phi_{0}\in (0,2\pi )$:

(47) $$r=\left\{\begin{array}{c} a\frac{sin(\phi_{0})}{\phi_{0}}\;=>\;\phi_{0}\neq 0\\ \theta \;=>\;\phi_{0}=0\; \end{array}\right.E_{pf}=\frac{\bar{v^{3}}}{{\bar{v}}^{3}}=\frac{\sum_{i=0}^{n}f_{i}v_{i}^{3}}{{\left(\sum_{i=0}^{n}f_{i}v_{i}\right)}^{3}}=\frac{\Gamma \left(1+\frac{3}{k}\right)}{\Gamma^{3}\left(1+\frac{1}{k}\right)}$$

where *θ* represents random environmental conditions sampled in [0, 1]. The foxes’ updated positions in multi-dimensional space are then expressed as:

(48) $$\left.\begin{array}{c} x_{1}^{new}=a\cdot r\cdot cos\left(\phi_{1}\right)+x_{1}^{old}\;\\ x_{2}^{new}=a\cdot r\cdot sin\left(\phi_{1}\right)+a\cdot r\cdot cos\left(\phi_{2}\right)+x_{2}^{old}\;\\ \begin{array}{c} x_{3}^{new}=ar\cdot sin\left(\phi_{1}\right)+ar\cdot sin\left(\phi_{2}\right)+ar\cdot cos\left(\phi_{3}\right)+x_{3}^{old}\\ \ldots \;.\\ x_{n}^{new}=a\cdot r\;\sum_{k=1}^{n-1}sin\left(\phi_{k}\right)+ar\cdot cos\left(\phi_{n}\right)+x_{n}^{old}\; \end{array} \end{array}\right\}$$

At each iteration, 5% of the worst-performing individuals are eliminated, simulating natural mortality. To maintain population size, new individuals are generated around the alpha pair’s habitat:

(49) $${\left(x_{repreduce}\right)}^{t}=\kappa \frac{x_{1}^{t}+x_{2}^{t}}{2}$$

(50) $$\kappa =\left\{\begin{array}{c} \kappa <0.45\;Reproduction\;of\;the\;pair\;\\ \kappa \geq 0.45\;Nomadic\;dispersal. \end{array}\right.$$

**Figure A6:**
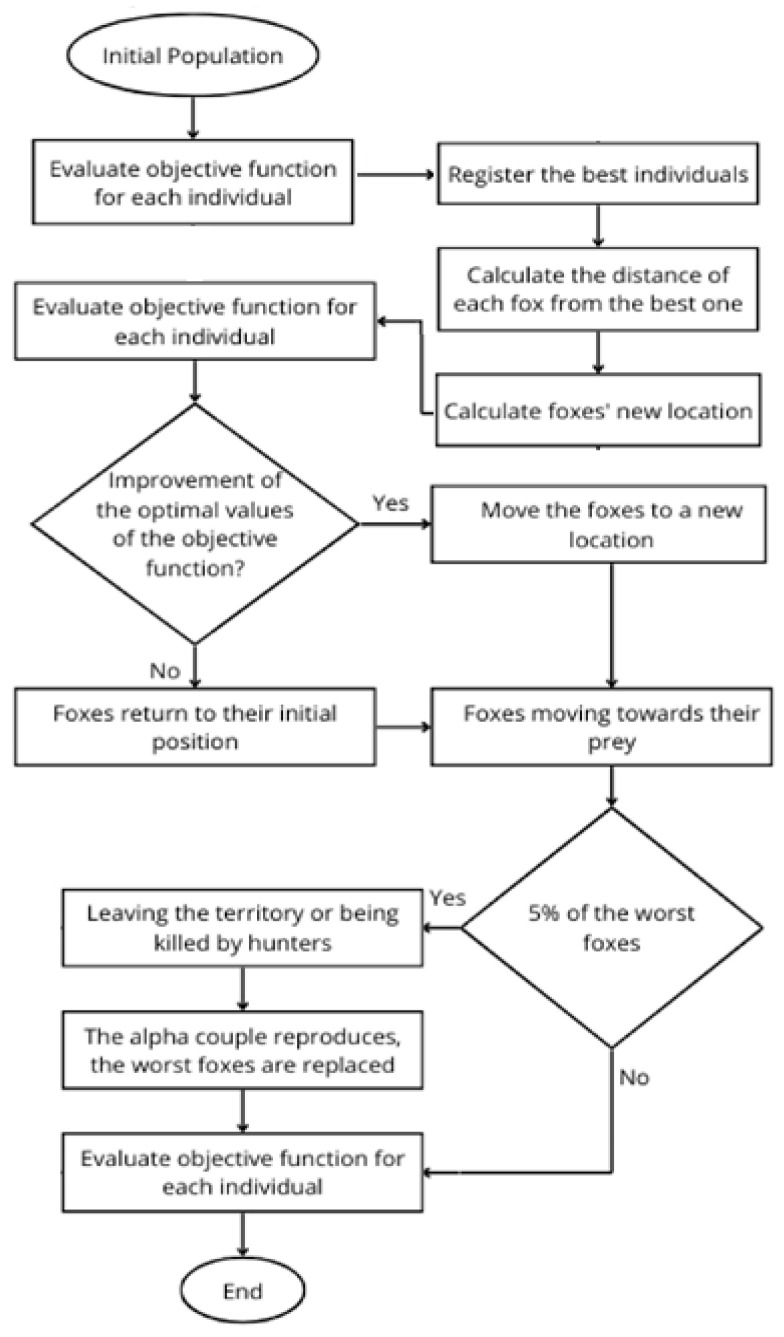
RFA flowchart [43].

Through this iterative process of global exploration, local exploitation, elimination, and reproduction, the RFA ensures both diversity and convergence, making it a robust optimization tool capable of achieving effective and balanced solutions in complex problem spaces [42,43]. The behavior of the red fox optimization algorithm in finding the optimal solution is shown in Figure A6 as a flow diagram.

#### 2.5.7. Red Panda Optimization Algorithm (RPA)

The Red Panda Optimization Algorithm (RPA) is a population-based metaheuristic inspired by the natural behaviors of the red panda, a small mammal native to southern China and the eastern Himalayas. Red pandas are characterized by their reddish-brown fur, black legs and abdomen, ringed tails, and distinctive white markings on the ears and face. Their flexible joints and semi-retractable claws allow excellent climbing ability, enabling them to thrive in temperate broadleaf, mixed, and coniferous forests, often near water sources with dense bamboo covers. As predominantly arboreal and solitary animals, red pandas are largely nocturnal, foraging at night using their acute vision, olfactory, and auditory senses, complemented by long whiskers that enhance their perception in low-light environments. Their foraging strategy, climbing behavior, and strong sensory capabilities form the biological foundation upon which the RPA is mathematically modeled [44].

In the RPA, each red panda is treated as a candidate solution in the search space, represented by the following population matrix:

(51) $$X={\left[X_{1},\ldots ,X_{i},\ldots ,X_{N}\right]}^{T}$$

where $X_{i}$ denotes the i-th panda and $x_{ij}$ represents its j-th decision variable. The initial population is generated randomly within the defined search boundaries:

(52) $$x_{ij}=lb_{j}+r_{ij}\left(ub_{j}-lb_{j}\right),\;r_{ij}\in [0,1]$$

Each candidate solution is then evaluated through the objective function:

(53) $$F\;=\;{\left[F\left(X_{1}\right),\;\ldots ,\;F\left(X_{i}\right),\;\ldots ,\;F\left(X_{N}\right)\right]}^{T}$$

The exploration phase models the food-searching behavior of red pandas. Pandas with better objective function values are treated as potential food sources:

(54) $$PFS_{i}=\left\{\;X_{k}\;\right|F_{k}\;<\;F_{i}\}\cup \;\{X_{best}\}$$

The new position of a panda is updated as:

(55) $$x_{ij}^{P1}\;=\;x_{ij}\;+\;r\cdot \;(SFS_{ij}\;-\;I\cdot \;x_{ij})$$

If the new position improves the objective function, the panda retains the new position:

(56) $$X_{i}=\left\{\begin{array}{c} X_{i}^{P1},\;F_{i}^{P1}\;<\;F_{i}\\ X_{i}\;aksi\;halde \end{array}\right.$$

In this phase, the resting behavior of red pandas, typically climbing trees, is mathematically modeled to represent local search and intensification. The new position is calculated as:

(57) $$x_{ij}^{P2}\;=\;x_{ij}\;+\;\frac{{lb_{j}+\;r}_{ij}\left(ub_{j}-lb_{j}\right)}{t}$$

where *t* denotes the iteration counter. The position is updated if the new solution yields a better objective value:

(58) $$x_{ij}^{P2}\;=\left\{\begin{array}{c} X_{i}^{P2},\;F_{i}^{P2}\;<\;F_{i}\\ X_{i}\;aksi\;halde \end{array}\right.$$

**Figure A7:**
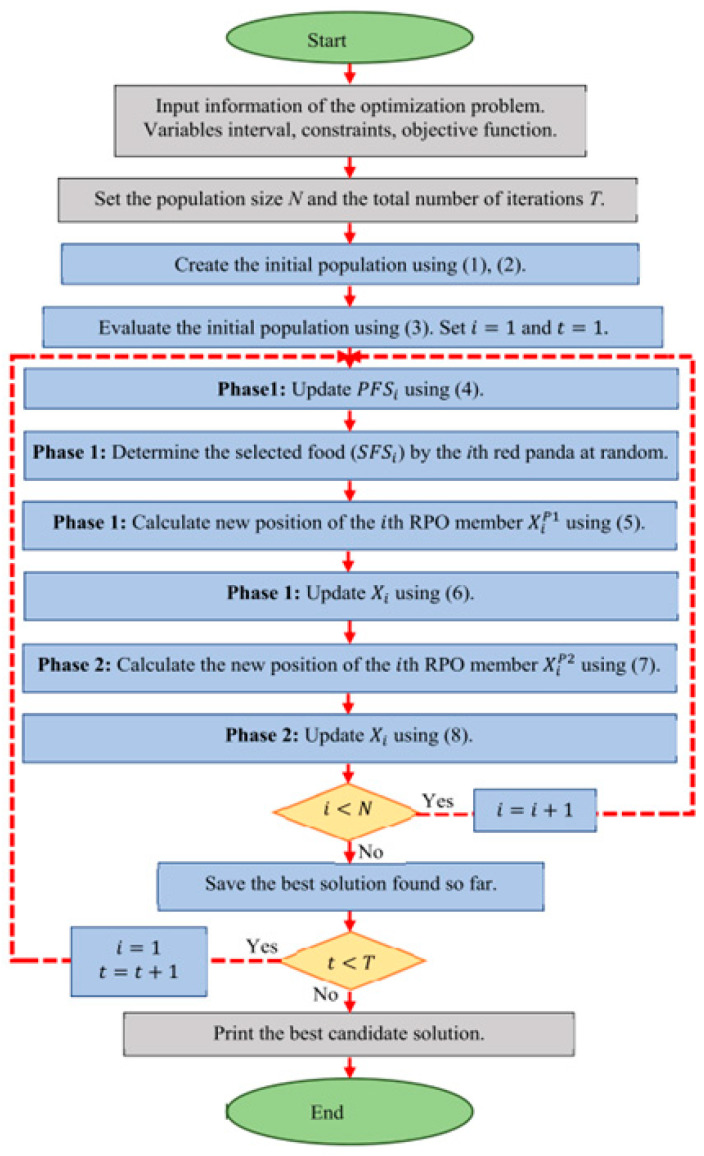
The flowchart for RPA [44].

Through these iterative exploration and exploitation stages, the RPA achieves a balance between global search (diversity) and local refinement, enabling effective convergence toward optimal solutions [44]. The behavior of the red panda optimization algorithm in finding the optimal solution is shown in Figure A7 as a flowchart.

### 2.6. On the Hub Heigth Tower Wind Power

Accurate determination of wind speed at the operating height of a wind turbine is crucial for ensuring efficient energy production. However, wind speed generally increases with altitude above ground level, and therefore the measurements obtained from meteorological masts or ground-level stations do not directly represent the wind conditions at the turbine hub height. To address this, a mathematical relationship known as the power law is applied, which establishes a link between wind speeds measured at the reference height and those at the turbine hub height. This enables a more precise estimation of wind speed at the actual operating level of the turbine

(59) $$V=V_{o}\;{\left(\frac{h}{h_{o}}\right)}^{\alpha }$$

Equation (59) expresses the power law, where *V* and $V_{0}$ denote the wind speeds at the turbine hub height and the reference (measurement) height, respectively. Here, ℎ is the hub height of the turbine, and $h_{0}$ is the measurement height of the station. The exponent *α*, referred to as the surface roughness coefficient, is a critical parameter that reflects the influence of terrain characteristics and obstacles on wind flow. By incorporating *α*, the model accounts for the interaction of wind with surface features, thereby providing a reliable extrapolation of wind speeds to hub height [20,45].

### 2.7. Wind Energy Indicators

Wind energy sites are typically assessed based on a range of technical and economic criteria, including turbine power rating, cost, hub height, design characteristics, and the wind regime of the site. Among the most critical indicators are the average wind speed, average power density, annual energy production, and the capacity factor of the turbines installed at the site

If $V_{i}$ denotes the average wind speed over the interval from $t_{i}$ to $t_{i}+∆t$, the average wind power generated by a turbine during *N* observation periods can be expressed as given in [19]:

(60) $$P_{w}=\frac{1}{N}\sum_{i=1}^{N}P(V_{i})$$

Under the same assumptions, the total energy output is calculated by:

(61) $$E_{out}=\sum_{i=1}^{N}P(V_{i})∆t$$

Alternatively, the cumulative energy output over a given period T may be written as:

(62) $$E_{out}=T\int_{v_{in}}^{v_{out}}P\left(V\right)\cdot f\left(V\right)dV$$

where f(V), denotes the Weibull probability density function (PDF), $P_{r}$ is the turbine’s rated power, $v_{in}$, $v_{r}$ and, are the cut-in, rated, and cut-out wind speeds, respectively. The capacity factor (*CF*), a key metric for turbine efficiency, is determined as:

(63) $$CF=\frac{E_{out}}{E_{rated}}\;$$

with the rated energy output defined as:

(64) $$E_{rated}\;=\;T\;\times P_{rated}$$

and for a turbine lifetime:

(65) $$E_{rated}=(n\;years)(365\;day)(24\;h)(P_{rated})$$

Thus, the total energy output is directly related to the capacity factor:

(66) $$E_{out}\;=\;CF\times \;E_{rated}$$

Average wind speed, which is central to wind energy potential assessment, is defined as the arithmetic mean of long-term wind speed measurements over a given period (e.g., 1 min, 10 min, hourly, daily, monthly, or annually). The accuracy of feasibility assessments depends heavily on both the duration and reliability of the recorded data.

Wind power density (WPD) is another fundamental parameter, representing the amount of energy available per unit area of the rotor swept surface. It is proportional to the cube of wind speed and serves as a key factor in site selection for wind energy projects. WPD is typically estimated using Weibull or Rayleigh distributions and is crucial for determining turbine efficiency and expected energy yield. The expression for WPD is:

(67) $$WPD=\frac{P_{w}}{A}=\int_{0}^{\infty }0.5\cdot \rho \cdot V^{3}\cdot f_{w}\left(V\right)dV=0.5\rho c^{3}\Gamma \left(1+\frac{3}{k}\right)$$

The annual energy production (AEP) can then be expressed as:

(68) $$AEP=\frac{CF\;\times \;A\;\times \;WPD\cdot 8760}{1000}\;(kWh)$$

The indicators described—average wind speed (AWS), wind power density (WPD), annual energy production (AEP), and capacity factor (CF), are fundamental for assessing the technical and economic viability of wind farm projects (Table 3). Among these, capacity factor (CF) is one of the most critical, as it directly reflects how effectively a turbine converts the available wind resource into electricity relative to its rated capacity. Higher CF values generally indicate more stable and economically attractive sites, reducing the risk of underperformance in long-term energy production.

Similarly, wind power density (WPD) provides a measure of the energy content of the wind resource per unit area, enabling comparisons across sites with different characteristics. A higher WPD not only suggests stronger wind regimes but also supports the deployment of larger turbines with higher hub heights and rotor diameters. When combined with annual energy production (AEP) calculations, investors can estimate expected revenues and compare alternative sites or turbine technologies. Together, these indicators form a decision-making framework that balances technical feasibility with financial considerations, guiding developers toward sites that maximize both energy yield and economic return.

### 2.8. Model Evaluation Criteria

The foregoing methods were used to estimate the frequency distribution of hourly average wind speeds and the parameters of Wpdf. The square root of the mean square error (RMSE) and R^2^ performance values are shown in Table 4 to evaluate the agreement between the estimated parameters and the frequency values generated using Wpdf and the real frequency. Equations (24) and (25) are used to compute the performance criterion and R^2^ [6,47].

(69) $$RMSE=\sqrt{\frac{1}{n}\sum_{i=1}^{n}{\left(V_{ci}-V_{oi}\right)}^{2}}$$

(70) $$R^{2}=1-\frac{\sum_{i=1}^{n}{\left(V_{ci}-V_{oi}\right)}^{2}}{\sum_{i=1}^{n}{\left(V_{oi}-\bar{V_{o}}\right)}^{2}}$$

*V_i_* is the *i*-th experimental data. $\bar{Vo}$ is the avarage wind speed value of the experimental data. The *i*-th estimated data by the founded Weibull disturbance is *V_ci_*, and the number of measurements is *n*.

The RMSE value represents the difference in estimated and measured values. The RMSE value must be as close to zero as possible [47].

### 2.9. Economic Analysis of the Station for Wind Energy

The economic evaluation of wind energy projects at observation station locations is critical for assessing the long-term financial feasibility of candidate wind turbines. Several factors influence overall project costs, including the initial investment, site-specific characteristics, and operational conditions. Turbine prices are generally determined by manufacturers according to the rated power, and for units larger than 200 kW, costs typically range between 1000 and 1600 USD per installed kilowatt. In this study, the Present Value Cost (PVC) method was employed to estimate the lifetime cost of wind energy production at four different sites in İzmir, Turkey. The PVC is calculated using Equation (71):

(71) $$PVC=I+C_{omr}\left[\frac{1+i}{r-i}\right]\cdot \left[1-{\left(\frac{1+i}{1-i}\right)}^{n}\right]-S{\left[\frac{1+i}{1+r}\right]}^{n}$$

where *I* denotes the initial investment, including turbine cost and additional civil works; $C_{omr}$ represents the annual O&M costs, *i* and *r* are the inflation and interest rates, respectively; *n* is the economic lifetime of the turbine (in years); and *S* denotes the salvage value [19]. Finally, the unit cost of electricity (UCE), expressed in $/kWh, is computed as:

(72) $$UCE=\left(\frac{PVC}{AEP}\;\frac{1\;\$}{1\;kWh}\right)$$

where *AEP* is the annual energy production. This metric allows direct comparison of alternative investment options by standardizing costs relative to energy output

In the literature, the standard life cycle of 20 years is widely used for the economic analysis of wind energy plant (WEP) projects. The determination of interest/discount rates depends on the economic conditions and risk profile of the country where the project is located. For example, [48] Jordan 8% Interest Rate Assumption: An 8% interest rate or discount factor has been used as a standard assumption in technical and economic analyses in Jordan. Similarly, reference [49] also uses an 8% discount factor in its economic analysis.

In reference to [50], it is assumed that the annual operating and maintenance cost is 2% of the wind turbine cost. [51], annual operating and maintenance costs were taken as 5% of the total investment cost. In this study, O&M costs are assumed to be 2.5% per year. These rates are based on general estimates in the industry and may vary from project to project or by geography. Scrap value is usually calculated as a percentage of the initial investment cost. In this study, the scrap value is assumed to be 10% of the investment cost and the turbine life is assumed to be 20 years.

It is noted that global average wind turbine prices ranged from USD 910–1050/kW in 2017 to USD 790–900/kW in 2018, and this downward trend is used in economic assessments [52,53].

The assumptions specified (20-year lifespan, 10% scrap value, and 8% interest rate) are supported by relevant studies in the literature and form the basis of these cost models. Such an approach is especially useful in identifying the most cost-effective turbine technology for a given location and provides a reliable decision-making framework for investors.

Table 4 presents the technical specifications of the wind turbines, including hub height, rotor diameter, cut-in and cut-out wind speeds, and rated power, which are critical parameters for investment decision-making. Notably, the inclusion of Present Value of Cost (PVC) values provides a robust basis for economic assessments, enabling the evaluation of turbine options not only in terms of energy production capacity but also in terms of cost-effectiveness. In the next section further details the annual energy outputs, capacity factors, cost of energy (COE), and payback periods across the four sites, offering a clear comparative perspective on the wind potential and investment feasibility of each location. For instance, while Foça and Karaburun exhibit relatively higher capacity factors, Çeşme stands out with low energy production and excessively long payback periods, underscoring significant regional disparities in investment attractiveness.

**Table 4:** Wind turbine technical aspects [52,53].

Turbine Technical Aspects	Bonus B23/150 kW	Halbes 315	Bonus B37/450 kW	Bonus B44/600 kW	Enercon E48-810 kW	Suzlon 1250 kW	Simens 2300 kW	Nordex 2.4 MW
	WT1	WT2	WT3	WT4	WT5	WT6	WT7	WT8
Hub height (m)	23	35	42	44	48	74	99	91
Rated power (kW)	150	315	450	600	810	1250	2300	2400
Rotor diameter (m)	23	35	42	44	48	66	113	116
Cut-inspeed (m/s)	4	2	4	3	2	4	3	3
Ratedspeed (m/s)	13	11	13	15	14	12	12	12
Cut-offspeed (m/s)	25	25	25	25	20	20	25	20
Price (US$)	180,000	400,000	480,000	500,000	850,000	1,395,000	2,350,000	2,450,000
PVC (US$)	76,366,888	169,704,195	203,645,034	212,130,244	360,621,416	591,843,382	997,012,150	1,039,438,199

When the turbine classes are compared with other technical characteristics, it is observed that the rotor diameters are generally consistent with their IEC classification, ranging from 23 m to 116 m. The turbines typically start operating at wind speeds between 2 m/s and 4 m/s, while their rated wind speeds range from 11.5 m/s to 15 m/s, and cut-out speeds are generally within 20 m/s to 25 m/s. According to the IEC 61400-1 standard, turbines are classified by manufacturers as Class I, II, III or Ia, IIa, IIIa, depending on their structural and operational characteristics. In this study, eight wind turbines with capacities ranging from 150 kW to 2.4 MW, various rotor diameters, and hub heights were selected to represent different scales of technology applicable to potential sites in the İzmir region. Rather than focusing on a single turbine type, the objective was to enable a comparative evaluation of diverse technologies across varying site conditions. According to the IEC 61400 standard, wind turbine classes are defined based on annual mean wind speed, extreme gusts, rotor diameter, and structural load capacity, which collectively determine suitability for specific wind regimes and ensure structural reliability over the turbine’s lifetime. Turbines such as Siemens 2300 kW and Bonus B44/600 kW, with higher rated wind speeds and robust designs, correspond to IEC Class I–IIA, making them ideal for high-wind sites. In contrast, Enercon E48-810 kW and Nordex 2.4 MW, characterized by larger rotors and low cut-in speeds, align with IEC Class IIIA, suited for low-to-medium wind conditions. Classification-based selection maximizes energy yield, annual energy production (AEP), and capacity factor (CF), while improving techno-economic performance across diverse wind regimes [54,55,56].

A comprehensive evaluation of both technical and economic parameters adds originality and practical relevance to the study. The comparative analysis of empirical methods and artificial intelligence–based optimization algorithms does not merely facilitate the selection of the most suitable turbine but also provides a decision-support framework for future investments. This dual approach elevates the study beyond theoretical research, positioning it as a practical roadmap for energy investors.

## 3. Results and Discussion

The wind speed distributions and fundamental statistical indicators for each site were derived from the recorded datasets, which provide insights into the frequency of wind occurrences at various intervals. This analysis offers a deeper understanding of the wind characteristics and their statistical behavior across different locations.

The frequency distribution of hourly mean wind speeds was modeled using the two-parameter Weibull probability distribution function (Wpdf), and the estimated parameters obtained from the applied methods are presented in Table 5. It was observed that, with the exception of the Energy Pattern Factor Method (EPFM), the estimated shape parameter *k* values across the other methods remained relatively close, ranging between 1.43 and 2.18. This indicates a consistent representation of the wind speed variability across sites. On the other hand, the scale parameter *c* results obtained from optimization-based methods—excluding Çeşme—were found to vary between 5.6 and 7.4, highlighting site-specific wind energy potential and the relative strength of wind resources. The changes in the k and c parameters generated by different algorithms not only represent a statistical difference but also provide complementary physical information about the stability (in k) and average intensity (in c) of the wind regime. This demonstrates that the framework proposed in our study not only provides investors with accuracy but also provides rich information into the nature of the wind resource.

**Figure 5:**
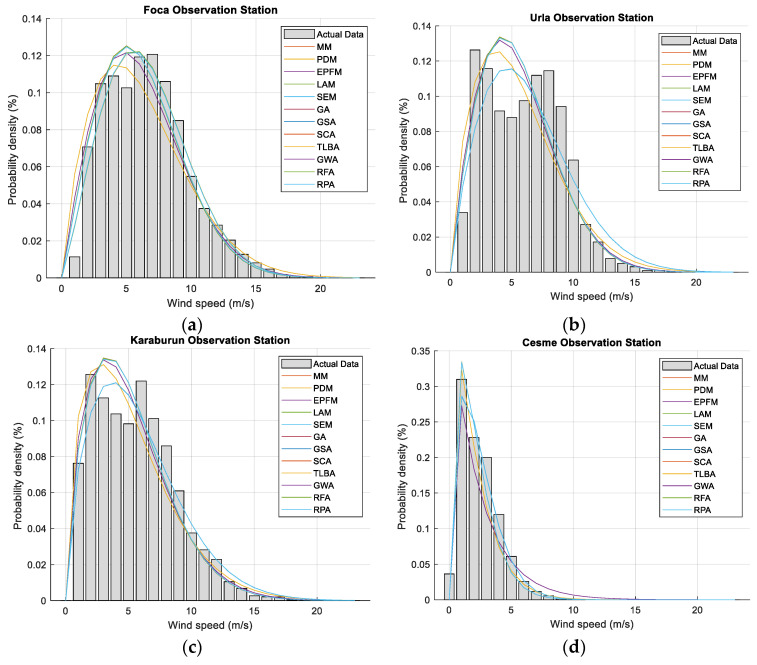
Speed distribution histograms at stations and frequency estimates of empirical and artificial intelligence-based algorithms.

Figure 5 presents the histograms of measured wind speeds at Foça, Urla, Karaburun, and Çeşme alongside Weibull distributions obtained using both empirical and optimization-based parameter estimation methods (e.g., MM, PDM, EPFM, GA, RF, RP). While histograms illustrate the empirical frequency distributions, overlaid curves demonstrate the degree of model fit. In Foça and Karaburun, metaheuristic algorithms (GA, RF, RP) aligned more closely with observed data compared to empirical methods, whereas in Çeşme, deviations were more pronounced due to low average wind speeds and higher turbulence, underscoring the limitations of simpler approaches under complex wind regimes.

These findings underscore the importance of model comparison as outlined in the study design. While empirical methods offer computational simplicity and rapid estimations, artificial intelligence–based optimization algorithms demonstrate greater flexibility and adaptability, resulting in improved accuracy. The graphical results further highlight that the choice of parameter estimation technique is critical in influencing downstream metrics such as the capacity factor, annual energy production, and cost of energy. Performance metrics presented in Table 6 highlight these trends more explicitly.

While classical empirical methods (MM, PDM, EPFM, LAM, SEM) yielded reasonable results, their RMSE values were comparatively higher, indicating weaker fit. Conversely, optimization algorithms (GA, GSA, SCA, TLBA, GWA, RF, RP) consistently achieved lower RMSE values across all locations, confirming their superior alignment with real-world data. Determination coefficients ($R^{2}$) further supported these findings: empirical methods generally produced values between 0.83 and 0.96, whereas optimization-based approaches consistently exceeded 0.97. For instance, at Karaburun, GA, GSA, and SCA reached $R^{2}=0.9606$, while SEM lagged at 0.9365. This demonstrates that optimization algorithms provide greater predictive accuracy, a critical advantage for estimating capacity factors and annual energy production. Among these, GA and related metaheuristic techniques demonstrated particularly strong alignment with empirical distributions, highlighting their capacity for precise parameter estimation.

Furthermore, the inter-site comparison reveals that empirical methods perform less effectively at the Çeşme station, whereas optimization algorithms maintain high predictive power even under complex wind regimes.

Overall, the results confirm that artificial intelligence–driven optimization techniques outperform traditional empirical approaches in modeling wind speed distributions. For investors, prioritizing methods with superior RMSE and $R^{2}$ performance can lead to more accurate forecasts of capacity factors and lower-cost energy production decisions. Moreover, the adaptability of metaheuristic algorithms across diverse geographic and meteorological conditions underscores their growing importance in sustainable energy planning and investment strategies. Table 7 gives a comparation of previous methods and our work, particularly in terms of innovation, algorithms used, and performance.

In terms of capacity factor, Foça and Urla demonstrate significantly higher values compared to the other sites, reflecting their stronger alignment between installed capacity and energy potential. Specifically, the Siemens 2300 kW (WT7) turbine achieved the highest capacity factor at Foça (42%), followed by the Nordex 2.4 MW (WT8) at 37%. In Urla, the Siemens 2300 kW reached 38%, while the Nordex 2.4 MW achieved 33%. Similarly, Karaburun recorded its highest capacity factor (32%) with the Siemens 2300 kW. By contrast, Çeşme displayed capacity factors as low as 2–4%, rendering it unsuitable for wind turbine deployment. These differences highlight the direct influence of regional wind regimes on energy generation potential.

With respect to annual energy production, high-capacity turbines unsurprisingly dominate. At Foça, the Siemens 2300 kW and Nordex 2.4 MW produced approximately 8.54 million kWh and 7.87 million kWh, respectively. In Urla, these turbines generated 7.66 million kWh and 6.93 million kWh, while in Karaburun they achieved 6.54 million kWh and 5.96 million kWh. Conversely, all turbines in Çeşme performed poorly, with the Siemens 2300 kW generating only 825,000 kWh—the highest output at that site. Economic indicators corroborate these technical results: while Foça and Urla recorded unit energy costs in the range of 0.0143–0.0184 $/kWh and short payback periods of 5.7–7.4 years, Çeşme showed extremely high COE values (0.1484–0.2060 $/kWh) and impractical payback times (59–235 years). Annual revenue analyses reflected similar trends, with the Siemens 2300 kW producing approximately 427,000 USD at Foça but only 41,000 USD at Çeşme.

The results presented in Table 8 clearly demonstrate that Foça and Urla are the most favorable sites for investment, offering high energy output, low COE, and short payback periods. Karaburun presents a moderately viable option, while Çeşme, characterized by low-capacity factors, high costs, and excessively long payback periods, is unsuitable for wind energy development. Overall, the Siemens 2300 kW and Nordex 2.4 MW turbines consistently delivered superior technical and economic performance across all sites, reinforcing the study’s contribution as a practical and reliable investment guideline for wind energy stakeholders.

The findings of this study highlight a clear link between statistical modeling accuracy and economic feasibility in wind energy investments. Optimization algorithms, particularly GA, GSA, and SCA, demonstrated superior predictive accuracy (low RMSE and $R^{2}$ high compared to classical empirical methods, enabling more reliable estimation of capacity factors and annual energy production. These improvements directly translated into robust techno-economic outcomes: in Foça and Urla, high-capacity turbines such as Siemens 2300 kW and Nordex 2.4 MW achieved both favorable payback periods (5.7–7.4 years) and competitive unit energy costs (<0.018 $/kWh). Conversely, the limitations of empirical methods were most evident in Çeşme, where poor model fit corresponded with low-capacity factors, high unit costs (0.148–0.206 $/kWh), and impractically long payback times (59–235 years). This direct alignment between accurate parameter estimation and realistic economic outcomes confirms that advanced metaheuristic optimization techniques not only improve statistical representation of wind regimes but also provide a decisive advantage in guiding sustainable, cost-effective investment decisions.

## 4. Conclusions

The comparative analysis of technical and economic indicators across the four wind monitoring sites (Foça, Urla, Karaburun, and Çeşme) reveals substantial regional variability in wind energy investment feasibility. Foça and Urla demonstrate the highest potential, achieving capacity factors of up to 42% (Siemens 2.3 MW) and annual energy yields exceeding 8 million kWh, accompanied by competitive unit energy costs (0.0143–0.0184 $/kWh) and relatively short payback periods (5.7–7.4 years). Karaburun exhibits moderate potential, while Çeşme is largely unsuitable for investment due to low capacity factors, high costs, and impractically long payback periods.

The superior performance of high-capacity turbines such as the Siemens 2.3 MW and Nordex 2.4 MW underscores the importance of turbine selection in maximizing energy output and economic viability. Furthermore, the integration of empirical methods with artificial intelligence-based optimization algorithms enhances the accuracy and reliability of Weibull parameter estimation, thereby improving the robustness of wind energy potential assessments.

From a methodological perspective, the results clearly demonstrate the superiority of artificial intelligence optimization algorithms (AIOAs) over empirical methods. For instance, the Genetic Algorithm (GA) reduced RMSE by approximately 21% compared to the best empirical method (SEM) and improved R^2^ values by nearly 4%. Similarly, other AIOAs (GSA, SCA, TLBO, GWO) achieved consistent accuracy, with RMSE as low as 0.0071 and R^2^ as high as 0.9755, whereas empirical methods such as PDM and EPFM showed higher errors (RMSE > 0.013, R^2^ < 0.95). These improvements indicate that AI-based methods not only enhance statistical reliability but also provide a more robust foundation for energy yield and techno-economic predictions.

Beyond these site-specific findings, the study highlights broader implications for sustainable wind energy deployment. These results can be used to prioritize turbine configurations to economic return. Future research should extend this framework to in-corporate dynamic climate projections, multi-year wind variability, and grid integration constraints, enabling and adaptive planning strategies. Additionally, the continued development of hybrid optimization approaches could further refine predictive accuracy and inform large-scale renewable energy policy and investment decisions.

## Figures and Tables

**Table 1:** Descriptive statistics of wind speed observations for the study sites.

Descriptive Statistic	Foça (Azaplar)	Urla	Karaburun	Çeşme
Mean (m/s)	6.1546	5.5214	5.1193	1.9833
Median (m/s)	5.9000	5.5000	4.9000	1.6000
Standard Deviation (m/s)	3.1860	3.0368	3.1672	1.5101
Kurtosis	0.2769	−0.4580	0.1339	1.1554
Skewness	0.6380	0.3463	0.6585	1.0810
Range (m/s)	21.5000	17.9000	18.9000	9.8000
Maximum (m/s)	21.9000	18.2000	19.1000	9.8000
Minimum (m/s)	0.4000	0.3000	0.2000	0.0000
Number of Observations	8594	8710	8107	8718

**Table 2:** Observation stations wind speed probability density.

Wind Speed (m/s)	Foça (Azaplar)	Urla	Karaburun	Çeşme
0	0.0000	0.0000	0.0000	0.0364
1	0.0113	0.0338	0.0762	0.3099
2	0.0707	0.1263	0.1257	0.2282
3	0.1048	0.1158	0.1126	0.2001
4	0.1090	0.0917	0.1037	0.1196
5	0.1026	0.0879	0.0982	0.0611
6	0.1193	0.0975	0.1220	0.0259
7	0.1207	0.1119	0.1011	0.0118
8	0.1060	0.1145	0.0859	0.0055
9	0.0849	0.0942	0.0609	0.0013
10	0.0548	0.0638	0.0375	0.0001
11	0.0374	0.0272	0.0282	0.0000
12	0.0285	0.0172	0.0229	0.0000
13	0.0204	0.0077	0.0105	0.0000
14	0.0127	0.0051	0.0068	0.0000
15	0.0081	0.0031	0.0027	0.0000
16	0.0047	0.0009	0.0021	0.0000
17	0.0019	0.0010	0.0020	0.0000
18	0.0009	0.0002	0.0005	0.0000
19	0.0007	0.0001	0.0002	0.0000
20	0.0001	0.0000	0.0001	0.0000
21	0.0002	0.0000	0.0000	0.0000
22	0.0002	0.0000	0.0000	0.0000
23	0.0000	0.0000	0.0000	0.0000

**Table 3:** Wind energy indictors [46].

Abbreviation	Explanation	Formula
AWS	Average Wind Speed	$AWS=c\Gamma \left(1+\frac{1}{k}\right)$
CF	Capacity factor	$CF=\frac{E_{out}}{E_{rated}}\;$
WPD	Wind Power Density	$WPD=\frac{1}{2}\rho c^{3}\Gamma \left(1+\frac{3}{k}\right)$
AEP	Annual Energy Production	$AEP=\frac{CF\cdot A\cdot WPD\cdot 8760}{1000}\;$

**Table 5:** Wpdf parameters k and c obtained by using methods examined for observation stations.

Methods (Empirical/AI)	Parameters	Foça (Azaplar)	Urla	Karaburun	Çeşme
MM or JEM	k	2.0443	1.9141	1.6845	1.3445
	c	6.9471	6.2236	5.7339	2.1613
PDM	k	1.7669	1.6882	1.5073	1.1701
	c	6.9142	6.1852	5.6741	2.0942
EPFM	k	1.9545	1.8617	1.6234	1.0167
	c	6.9413	6.2178	5.7173	2.4620
LEM	k	2.0443	1.9141	1.6845	1.3445
	c	6.9508	6.2276	5.7384	2.1628
SEM	k	2.0510	1.9198	1.6885	1.3464
	c	6.9912	6.2596	5.7618	2.1688
GA	k	2.1609	1.8476	1.6660	1.4463
	c	7.3704	7.0389	6.3746	2.5682
GSA	k	2.1736	1.8501	1.6682	1.4487
	c	7.3760	7.0390	6.3755	2.5713
SCA	k	2.1608	1.8477	1.6664	1.4446
	c	7.3707	7.0392	6.3753	2.5649
TLBA	k	2.1676	1.8476	1.6663	1.4448
	c	7.3890	7.0389	6.3755	2.5647
GWA	k	2.1736	1.8477	1.6662	1.4448
	c	7.3760	7.0388	6.3756	2.5648
RFA	k	2.1736	1.8476	1.6663	1.4448
	c	7.3760	7.0390	6.3756	2.5648
RPA	k	2.1613	1.8475	1.6663	1.4449
	c	7.3707	7.0389	6.3757	2.5646

**Table 6:** Performance Values of Parameter Estimation Methods.

Methods	Metrics	Foça	Urla	Karaburun	Çesme
MM	RMSE	0.0093	0.0193	0.0119	0.0176
JEM	R^2^	0.9584	0.8348	0.9343	0.9555
PDM	RMSE	0.0140	0.0194	0.0132	0.0196
	R^2^	0.9059	0.8340	0.9203	0.9449
EPFM	RMSE	0.0104	0.0191	0.0120	0.0231
	R^2^	0.9485	0.8389	0.9334	0.9228
LAM	RMSE	0.0093	0.0193	0.0119	0.0175
	R^2^	0.9586	0.8353	0.9347	0.9557
SEM	RMSE	0.0090	0.0191	0.0117	0.0174
	R^2^	0.9615	0.8383	0.9365	0.9564
GA	RMSE	0.0071	0.0163	0.0093	0.0120
	R^2^	0.9755	0.8817	0.9606	0.9791
GSA	RMSE	0.0071	0.0163	0.0093	0.0120
	R^2^	0.9755	0.8816	0.9606	0.9791
SCA	RMSE	0.0071	0.0163	0.0093	0.0120
	R^2^	0.9755	0.8817	0.9606	0.9791
TLBA	RMSE	0.0071	0.0163	0.0093	0.0120
	R^2^	0.9755	0.8817	0.9606	0.9791
GWA	RMSE	0.0071	0.0163	0.0093	0.0120
	R^2^	0.9755	0.8817	0.9606	0.9791
RFA	RMSE	0.0071	0.0163	0.0093	0.0120
	R^2^	0.9755	0.8817	0.9606	0.9791
RPA	RMSE	0.0071	0.0163	0.0093	0.0120
	R^2^	0.9755	0.8817	0.9606	0.9791

**Table 7:** Comparative summary of empirical, established AI, and newly proposed algorithms in Weibull parameter estimation.

Algorithm Group	Algorithms	RMSE/R^2^ Range	Expectation in the Literature	Observation in This Study	Interpretation	Literature Evidence
Classical Empirical Methods	MM, PDM, EPFM, LAM, SEM	RMSE: 0.0090–0.0231, R^2^: 0.83–0.96	Sufficient but limited accuracy	Relatively higher RMSE and lower R^2^	Simplicity makes them less accurate in complex wind regimes.	Genç et al. [14]: Akşehir (Turkey), MM, MLM, LSM consistent but higher errors vs. AI; EPFM often reliable [17].
Established Metaheuristics	GA, GSA, SCA, TLBA, GWA	RMSE 0.0071–0.0163, R^2^: 0.88–0.98	High accuracy, fast convergence	Similar high performance across all sites	Metaheuristics have long been validated for Weibull parameter estimation.	Çatalca (Istanbul) [15]: GA vs. GM → GA superior; Egypt [16]: AO > PSO > classical; Alrashidi et al. [9]: multiple metaheuristics benchmarked, showing AI consistently outperforming empirical methods; Saeed et al. [20]: Pakistan, AI-enhanced Weibull fitting improved accuracy of economic predictions; Wan et al. [46]: China (Urat), optimized Weibull via metaheuristics improved fit and wind potential assessment.
Algorithms Proposed in This Study	Red Fox (RFA), Red Panda (RPA)	RMSE: 0.0071–0.0163, R^2^: 0.88–0.98	First applications, performance unknown	Performance equal to GA, GSA, SCA, TLBA, GWA	Indicates RFA and RPA are at least as effective as strong established methods, validating their reliability and offering methodological diversity for future applications.	No previous application of RFA and RPA in wind modeling reported in the literature.

**Table 8:** Wind energy investment indicators.

Site	Wind Turbine	Annual Energy Output (MWh/year)	Capacity Factor (%)	COE ($/kWh)	Payback Period (Years)
Foça	WT1	412,551	0.31	0.0227	9.10
	WT2	905,378	0.33	0.0230	9.21
	WT3	967,146	0.25	0.0259	10.35
	WT4	1,353,142	0.26	0.0193	7.70
	WT5	2,018,481	0.28	0.0219	8.78
	WT6	3,037,142	0.28	0.0239	9.58
	WT7	8,541,998	0.42	0.0143	5.74
	WT8	7,867,731	0.37	0.0162	6.49
Urla	WT1	354,967	0.27	0.0264	10.57
	WT2	781,287	0.28	0.0267	10.67
	WT3	808,996	0.22	0.0309	12.37
	WT4	1,140,039	0.24	0.0229	9.14
	WT5	1,709,839	0.23	0.0259	10.36
	WT6	2,569,092	0.23	0.0283	11.32
	WT7	7,659,254	0.38	0.0160	6.40
	WT8	6,932,698	0.33	0.0184	7.37
Karaburun	WT1	307,364.05	0.23	0.0305	12.21
	WT2	680,031.79	0.25	0.0307	12.26
	WT3	705,975.85	0.18	0.0354	14.18
	WT4	959,917.73	0.19	0.0262	10.47
	WT5	1,500,422.44	0.21	0.0295	11.81
	WT6	2,235,635.43	0.20	0.0325	13.01
	WT7	6,540,635.60	0.32	0.0187	7.49
	WT8	5,956,430.33	0.28	0.0214	8.58
Çeşme	WT1	27,689	0.02	0.3388	135.53
	WT2	80,399	0.03	0.2593	103.72
	WT3	42,542	0.01	0.5881	235.23
	WT4	80,661	0.02	0.3231	129.23
	WT5	159,635	0.02	0.2775	111.01
	WT6	182,685	0.02	0.398	159.2
	WT7	825,098	0.04	0.1484	59.38
	WT8	619,747	0.03	0.206	82.42

## Data Availability

The original contributions presented in this study are included in the article. Further inquiries can be directed to the corresponding author.

## Appendix A

In this section all flowcharts of the algorithms used in this study will be given in Figure A1, Figure A2, Figure A3, Figure A4, Figure A5, Figure A6 and Figure A7.
